# A Review of Recent Advances in Superhydrophobic Surfaces and Their Applications in Drag Reduction and Heat Transfer

**DOI:** 10.3390/nano12010044

**Published:** 2021-12-23

**Authors:** Yu Zhang, Zhentao Zhang, Junling Yang, Yunkai Yue, Huafu Zhang

**Affiliations:** 1 Technical Institute of Physics and Chemistry of CAS, Beijing 100190, China; zhangyu19d@mail.ipc.ac.cn (Y.Z.); zzt@mail.ipc.ac.cn (Z.Z.); yueyunkai@mail.ipc.ac.cn (Y.Y.); zhanghuafu@mail.ipc.ac.cn (H.Z.); 2 University of Chinese Academy of Sciences, Beijing 100049, China; 3 Key Laboratory of Food & Pharmaceutical Quality Processing Storage and Transportation Equipment and Energy-Saving Technology, China National Light Industry, Beijing 100190, China

**Keywords:** superhydrophobic surface, preparation, drag reduction, heat transfer, highly viscous fluid

## Abstract

Inspired by the superhydrophobic properties of some plants and animals with special structures, such as self-cleaning, water repellent, and drag reduction, the research on the basic theory and practical applications of superhydrophobic surfaces is increasing. In this paper, the characteristics of superhydrophobic surfaces and the preparation methods of superhydrophobic surfaces are briefly reviewed. The mechanisms of drag reduction on superhydrophobic surfaces and the effects of parameters such as flow rate, fluid viscosity, wettability, and surface morphology on drag reduction are discussed, as well as the applications of superhydrophobic surfaces in boiling heat transfer and condensation heat transfer. Finally, the limitations of adapting superhydrophobic surfaces to industrial applications are discussed. The possibility of applying superhydrophobic surfaces to highly viscous fluids for heat transfer to reduce flow resistance and improve heat transfer efficiency is introduced as a topic for further research in the future.

## 1. Introduction

**Figure 1:**
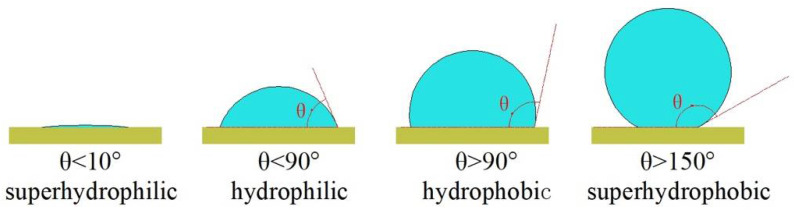
Droplet state on the surface.

**Figure 2:**
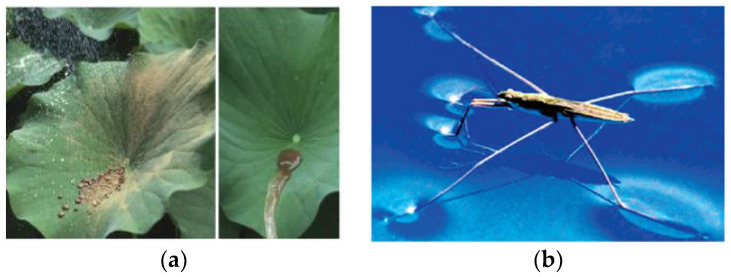
Superhydrophobic surface. (**a**) Lotus leaf. Reprinted with permission from Ref [43]. Copyright 2015 Elsevier. (**b**) Water strider. Reprinted with permission from Ref [3]. Copyright 2007 American Chemical Society.

**Figure 3:**
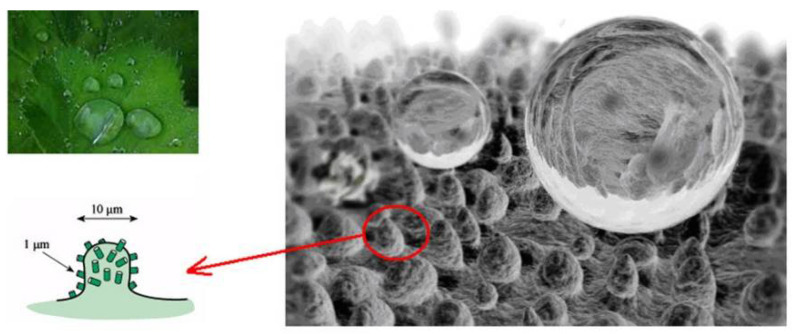
Superhydrophobicity and the multiscale structural surface of Lotus leaf. Reprinted with permission from Ref [44]. Copyright 2011 Elsevier.

The wettability of surfaces can be characterized by the contact angle (CA). Generally, the hydrophilic surface has a contact angle below 90°, and the contact angles below 10° are defined as superhydrophilic surfaces. The surfaces with contact angles above 90° are called hydrophobic surfaces, and the contact angle above 150° and the sliding angle below 5° are defined as superhydrophobic surfaces [1,2], as shown in Figure 1. The inspiration of the superhydrophobic surface comes from natural plants and insects such as lotus leaves, rice leaves, mosquito feet, butterfly wings, and water striders all show superhydrophobic properties like self-cleaning, water-repelling, easily rolling off the surface, as shown in Figure 2a, the water can completely roll off the lotus leaves without footprint remains. In Figure 2b, the water striders that are described as “skaters in a pond” can walk on water easily and fastly [3,4,5]. These particular characteristics are ascribed to the special binary micro/nanostructures and low surface energy on the plants and insects’ surfaces [5,6]. For instance, they are randomly distributed with micron and nanoscale level papillary on the lotus leaf (shown in Figure 3), and a layer of low surface energy cuticle wax is covered on the surface [7,8]. If the superhydrophobic features can be functionalized on various metal surfaces, it will be significant and beneficial in many industrial applications for saving energy and energy storage [9]. For example, it can drag reduction, anti-fouling, and enhance heat transfer performance.

**Figure 4:**
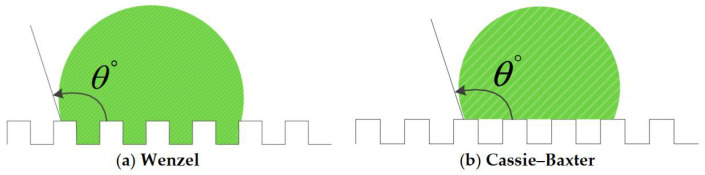
The states of droplets on roughness surface. (**a**) The Wenzel model. (**b**) The Cassie–Baxter model. Reprinted from Ref [45].

The contact angle is usually measured by a contact angle meter when a 2 µL or 5 µL water droplet rests on a surface. The CA is one of the most important parameters in characterizing the wettability of a surface. In 1805, British scientist Thomas Young first proposed the correlation between the CA and the surface tension on an ideally smooth surface. Since Young’s theory is strictly valid for ideally smooth surfaces, the Wenzel [10] model and Cassie–Baxter [11] model were established to introduce the surface wetting state of partial rough surfaces. Based on the Wenzel model, the water droplet completely penetrates the micro/nanostructures of the rough surface, as shown in Figure 4a, in which the increase of the surface roughness of a hydrophobic surface can enhance the static CA and make the surface more hydrophobic. Therefore, the superhydrophobic surface can be prepared by creating a rough structure on the hydrophobic surface. Wenzel’s conclusion was further studied by Cassie and Baxter; they considered the situation that the water droplet’s adhesion force of a particularly rough surface was not enough to make the surface completely wetting, so in their theory, the water droplet is suspended on the micro/nanostructures, and a discontinuous air cavity is formed between the droplet and the rough surface, as shown in Figure 4b. The discontinuous air cavities remarkably reduce the contact area of a water droplet and solid surface, which is resulted in a larger corresponding CA of Cassie–Baxter model than the Wenzel model, and the water droplet can easily roll off the surface. Cassie and Baxter studied the effect of the porous composite surface with different chemical components on the droplet contact angle. Cassie–Baxter equation is as follow:

(1) $$\cos\theta_{C}=f_{1}\cos\theta_{1}+f_{2}\cos\theta_{2}$$

where *f*_1_ and *f*_2_ are the area fraction of the liquid contacting components 1 and 2, and the corresponding intrinsic contact angles are *θ*_1_ and *θ*_2_, and *f*_1_ + *f*_2_ = 1. Swain et al. [12] investigated the wetting law on geometrically rough and chemically heterogeneous surfaces. A new complex equation is proposed for the structure of substrates with both geometric and chemical structures:

(2) $$\cos\theta_{e}=\sum_{i}r_{i}\left(\cos\theta_{i}-\frac{\lambda_{i}C_{i}}{\sigma }\right)+\Delta \rho g\bar{Z^{2}}$$

where *θ_e_* is the average contact angle taken up by the drop on a heterogeneous substrate, *r_i_* is the ratio of the non-planar area covered by the material to the total planar area, *σ* is the interfacial tensions, *λ* is the line tension, and $\bar{Z^{2}}$ is the mean square height of the substrate.

Both the micro-nano roughness structures and modification with low surface energy on the surface are the important factors influencing the superhydrophobicity of a surface, while superhydrophobic surface cannot be obtained only modified by low surface energy materials [13]. Therefore, both the surface roughness and the low surface energy are indispensable in order to obtain the superhydrophobic surface [14]. Based on this theory, scholars have developed a series of methods to fabricate superhydrophobic surfaces on various metal substrates, such as laser etching method [15], chemical etching [16,17], sol-gel method [18], chemical deposition method [19], vapor deposition method [20], template method [21], anodic oxidation [22], electrospinning method [23], and layer-by-layer assembly method [24].

With the development of manufacturing superhydrophobic surfaces on various metal substrates, these functionalized metal superhydrophobic surfaces with interesting characteristics are widely applied in multifarious industries and everyday life. For instance, the surface of metal pipelines is usually rough surface and hydrophilic; the fluids inside the pipes undergo enormous pressure drops, which causes tremendous energy consumption. Therefore, the superhydrophobic surface with drag reduction property is able to overcome this problem effectively [25]. Surface wettability plays a key role in boiling heat transfer, and the heat transfer performance of superhydrophobic surfaces increases significantly at small superheats due to the rapid removal of nucleated bubbles from the surface, improving the heat transfer coefficient. When a superhydrophobic surface is applied to a condensing heat exchanger tube, not only the pressure drop can be reduced, but the convective heat transfer performance is also improved to some extent [26]. The wind turbines and aircraft are usually working in hostile environments because of ice and snow accumulation and adhere to the surfaces, which causes catastrophic risks; thus, the superhydrophobic surfaces with anti-icing properties can avoid ice and snow accumulation and adhesion and enhance the efficiency [27]. Superhydrophobic meshes can be practically used in petroleum industries to separate oil and water [28]. Moreover, superhydrophobic surfaces have many other applications such as waterproof [29,30], self-cleaning [31,32,33,34], anti-frosting [35,36,37,38], and anti-corrosion [39,40,41,42].

The above content demonstrates that superhydrophobic surfaces have many useful potential applications in industries and everyday life, especially in the energy-saving field. This paper illustrates the fabrications and characteristics of superhydrophobic surfaces on the metal surface. The characteristics of drag reduction, boiling heat transfer, and condensation heat transfer on the superhydrophobic surface are illustrated. Finally, we discuss the feasibility of using the superhydrophobic surface to reduce the flow resistance and improve the heat transfer efficiency in the heat transfer process of the viscous fluid.

## 2. Fabrication of Superhydrophobic Surfaces

In industrial applications, the pipes, plates, and heat exchangers are usually manufactured by metals or alloys, such as stainless steel, aluminum, and copper. Thus, it is necessary to prepare the superhydrophobic surface on metal substrates. The process of fabricating superhydrophobic surfaces on metal substrates consists of two steps, firstly the fabrication of micro-nano roughness structures and then modifying the roughness surfaces with low surface energy chemical materials. Over the recent decades, scholars have developed various methods to fabricate superhydrophobic surfaces on metal substrates, such as the etching method, sol-gel, and electrochemical deposition; other fabrication methods are also described in the paper.

### 2.1. Etching 

The etching method is mainly inspired by plants and animals in nature. The etching method is a simple approach, and the main purpose is to increase the surface roughness of the metal substrates. After the surface micro-nano roughness structures are fabricated, a low surface energy material is then modified to the surface to obtain the superhydrophobic surface. Common etching methods include Laser ablation [46,47,48], plasma etching [49], and chemical etching [50,51,52].

#### 2.1.1. Laser Ablation

**Figure 5:**
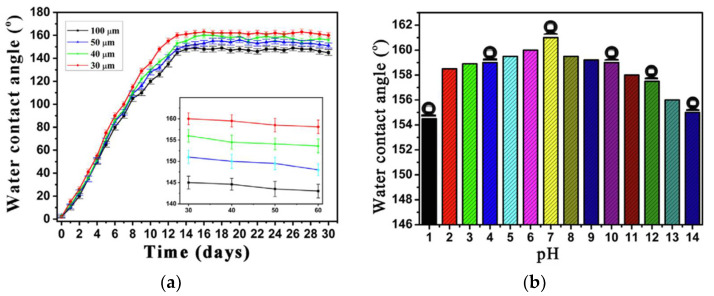
(**a**) Water contact angle for the time. (**b**) Water contact angle with different pH values. Reprinted with permission from Ref [53]. Copyright 2020 Elsevier.

**Figure 6:**
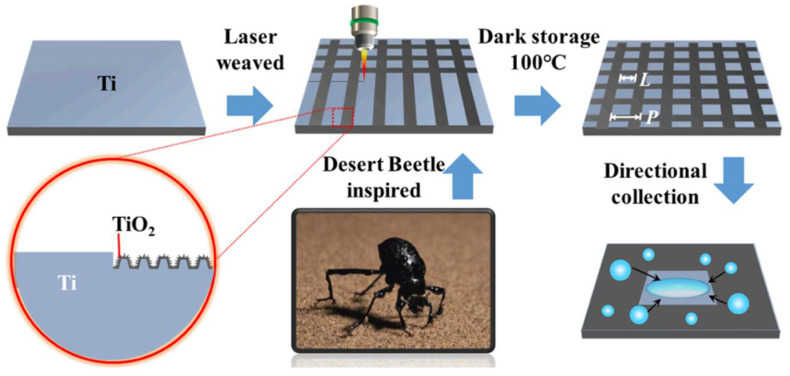
The schematic of femtosecond laser weaving process on a Ti surface. Reprinted with permission from Ref [54]. Copyright 2022 Elsevier.

**Figure 7:**
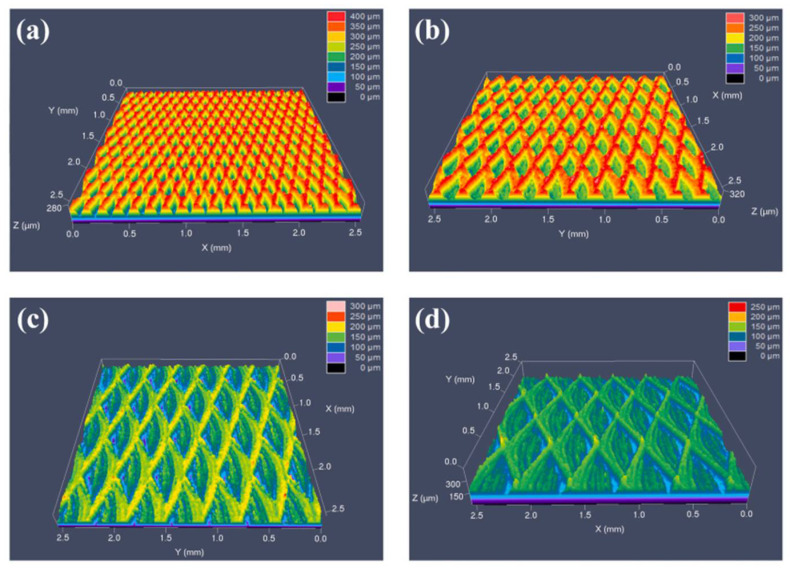
Micro-morphology of the bionic fish-scale surface under laser confocal scanning microscope. (**a**) Micro-morphology image of 30%-size fish-scale surface. (**b**) Micro-morphology image of 50%-size fish-scale surface. (**c**) Micro-morphology image of 80%-size fish-scale surface. (**d**) Micro-morphology image of 100%-size fish-scale surface. Reprinted with permission from Ref [55]. Copyright 2021 Elsevier.

The laser ablation technique has aroused great attention in the manufacture of superhydrophobic surfaces on the metal substrates because of the high precision, excellent ability to construct binary micro-nano structures, high automation, and environmental friendliness. In order to obtain the superhydrophobic surface, picosecond laser, femtosecond laser, and nanosecond laser have been applied. Qian et al. [53] fabricated a superhydrophobic surface with a contact angle greater than 163° and a sliding angle lee than 3° on nickel-aluminum bronze (NAB) surface by the picosecond laser method. The stability of the superhydrophobic surface was investigated by exposing the surface to atmospheric conditions for 14 days (Figure 5a), and the NBA surface still maintained high contact angle, which showed the stability superhydrophobicity. The corrosion test was conducted under an environment with different pH values, and the result showed (Figure 5b) the excellent anti-corrosion as the contact angles were around 154–164°. Inspired by desert beetles, femtosecond laser ablation was used to form superhydrophobic micro-patterns and TiO_2_ layer on Ti surfaces; the schematic is shown in Figure 6 after the femtosecond laser weaving process, the substrate was dark storage at 100 °C for 24 h, then the superhydrophobic surface with TiO_2_ microgroove and water contact angle of 157 ± 1° was obtained. Attributed to the formation of TiO_2_, the Ti surface is able to transfer from superhydrophilicity to superhydrophobicity [54]. Wang et al. [55] designed a bionic fish-scales surface based on the morphology of scales on the body of Sciaenops ocellatus, and the nanosecond laser processing method was adopted to fabricate a bionic fish-scale structure with four kinds of zoom ratios (30%, 50%, 80%, and 100%) on the Al alloy substrates. As shown in Figure 7, the oblique-like fish-scale groove-ridge structures fabricated on the Al alloy substrate were similar to the grooves’ structures of the real fish. If the scale pattern was less than 50%, the laser method was failed to fabricate a complete fish-like rough structure on the Al alloy substrate. However, when the zoom ratio was larger than 50%, regular and clear fish-scale grooves, microspheres, and arc-shaped groove-ridges were completely prepared on the Al alloy substrate. The complex surface with micro-nano structures can catch more air into the gap of the micro-nano structures, showing good superhydrophobicity, and the water contact angle was 154.9° when the zoom ratio was 100%.

#### 2.1.2. Plasma Etching

**Figure 8:**
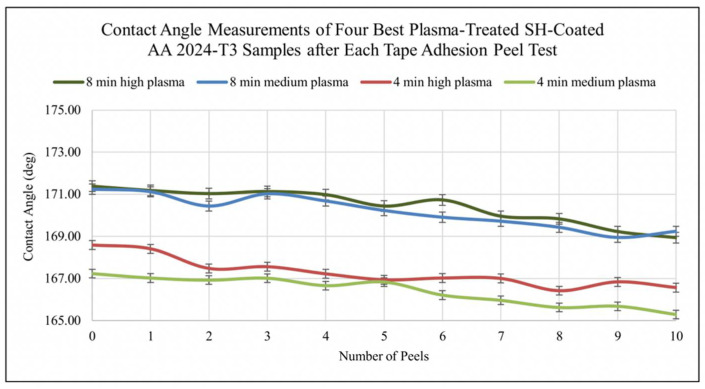
The contact angle after tape adhesion peel test. Reprinted with permission from Ref [57]. Copyright 2020 Elsevier.

**Figure 9:**
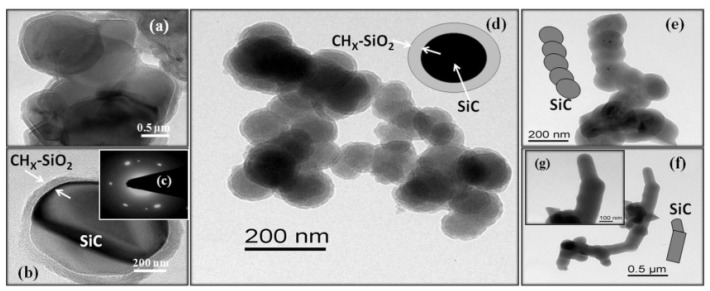
Electron scanning electron microscopy images. (**a**,**b**) Growth time of 1 min. (**c**) Diffraction pattern of the particle core. (**d**) Growth time of 2 min. (**e**) Beaded lines with a growth time of 3 min. (**f**,**g**) Smooth surface single lines with a growth time of 3 min. Reprinted with permission from Ref [59]. Copyright 2016 Elsevier.

The plasma etching method employs plasma processing with highly anisotropic conditions (plasma micro-nanofabrication) to create random or quasi-ordered rough structures on the surface. Several studies have successfully applied this method to fabricate superhydrophobic surfaces [56]. The major concern for deicing and self-cleaning metals and alloys can be easily solved by fabricating superhydrophobic coated surfaces. However, the weak bond between the surface and the coating makes the fabricated surfaces unable to withstand serious environmental changes. Therefore, how to produce highly robust superhydrophobic coatings on surfaces is the focus of research [57]. Subeshan et al. [57] reported the use of the plasma-treated process to fabricate the deicing and self-cleaning superhydrophobic coatings on the aluminum 2024-T3 alloy. The superhydrophobic surfaces were heat-treated at 150 °C for 60 min prior to deicing and self-cleaning process application. The plasma-treated coatings were tested for tape adhesion, Vickers microhardness (HV), deicing, and self-cleaning. The results showed that the plasma-treated superhydrophobic coating increased adhesion between the substrate and topcoat, provided a water contact angle of over 165°, and exhibited higher deicing and self-cleaning performance. The tape adhesion test results are shown in Figure 8, and the contact angle starts to decrease slightly with each peel test; however, the majority of the surface remains superhydrophobic level, also clearly demonstrating the robustness of the metal surface with the superhydrophobic coating. Vickers microhardness tests showed that the mechanical properties of the surfaces were not altered by the plasma surface cleaning process. Therefore, the heat treatment process after plasma treatment can develop robust superhydrophobic coatings. Sharifi et al. [58] used atmospheric plasma spraying (APS) and suspension plasma spraying (SPS) methods to fabricate superhydrophobic coatings with high water repellence and mobility on stainless steel substrates. The experimental results indicate that the coatings developed by SPS show excellent water repellency with contact angle up to 167° and roll angle 1.3° higher than 145° developed by APS. This is due to the use of a suspension containing submicron TiO_2_ particles, which creates a graded roughness and results in markedly improved water mobility. Sahoo et al. [59] described a thermal plasma technique for fabricating superhydrophobic surfaces on stainless steel substrates and optimized the process, resulting in a high-quality superhydrophobic coating with a contact angle of 150.8°. The presence of crystalline SiC cores and S-O-CH_X_ shell particles in the formed nanostructures was confirmed using transmission electron microscopy and X-ray diffraction patterns, as shown in Figure 9. They confirmed that the superhydrophobicity was attributed to the high surface area of the nanostructures and the surface functionalization of the silicon shell layer of the nanostructures formed in the coating.

#### 2.1.3. Chemical Etching

**Figure 10:**
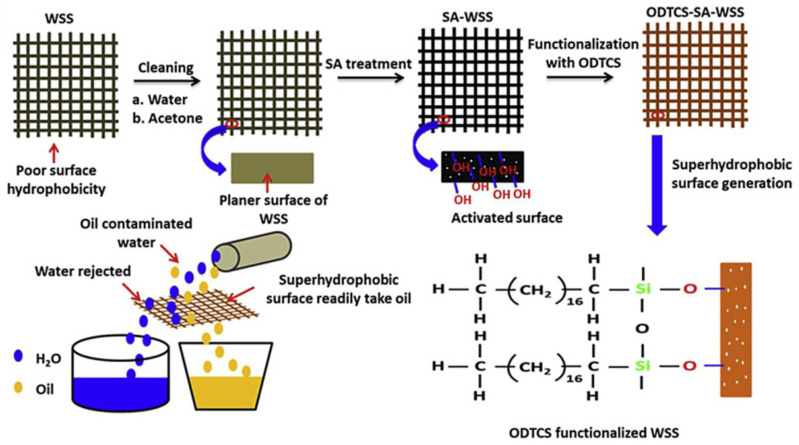
The development of the superhydrophobic surface fabrication for oil and water separation. Reprinted with permission from Ref [61]. Copyright 2019 Elsevier.

**Figure 11:**
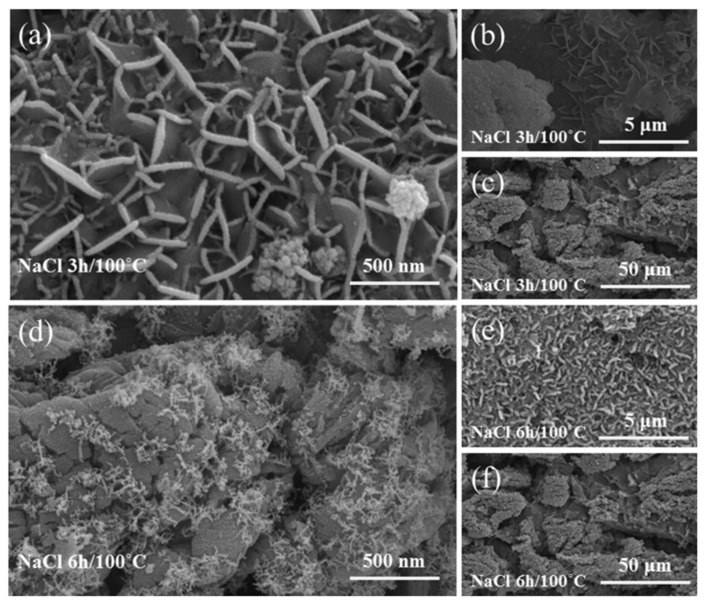
FE-SEM images of stainless-steel surface after dipping in NaCl solution. (**a**–**c**) dipped 3 h at different magnifications. (**d**–**f**) dipped 6 h at different magnifications. Reprinted with permission from Ref [62]. Copyright 2018 Elsevier.

Chemical etching is an inexpensive and simple approach method of immersing metal substrates into acid or base solution to create binary micro-nano structures; those solutions could be a strong acid, such as HCl and H_2_SO_4_, and a strong base, such as NaOH. Moreover, the technique is universal and can be applied to almost any metal or alloy [60]. After the surface roughness structures are created, low surface energy materials are then modified to the surface to fabricate a superhydrophobic surface. Saleh et al. [61] used sulfuric acid solution to immerse the stainless-steel mesh to construct roughness structures and oxidizing functional groups. Then, the low surface energy material octyltrichlorosilane (ODTCS) was used for surface functionalization to obtain a superhydrophobic and superoleophilic surface with a contact angle up to 166.8°, and the oil-water separation efficiency was more than 99% (Figure 10). Kim et al. [62] etched the austenitic stainless steel in an HF solution, followed by modification of low surface energy chemical material, then the superhydrophobic surface with a water contact angle of 166° and a sliding angle of 5° was obtained. To further enhance the superhydrophobicity of stainless-steel surface, the samples treated with HF solution were dipped in a 0.1 wt.% NaCl solution at 100 °C for 3 h and 6 h, the FE-SEM images of the results shown in Figure 11, a petal-like hierarchical microstructure is generated on the sample surface, the water contact angle and sliding angle after NaCl dipped were 168° and ~2°, respectively. Moreover, the further increases in dipping time did not affect the superhydrophobicity of the sample surface. The 304 stainless-steel surfaces with binary micro-nanoscale structures can be fabricated by chemical method with a mixture solution of ferric trichloride, hydrochloric acid, and hydrogen peroxide, followed by modifying the rough surface with low free energy material of DTS (C_6_H_5_CH_3_). Then, a superhydrophobic surface with a contact angle of 158.3 ± 2.8° was fabricated [63]. 

### 2.2. Sol-Gel

**Figure 12:**
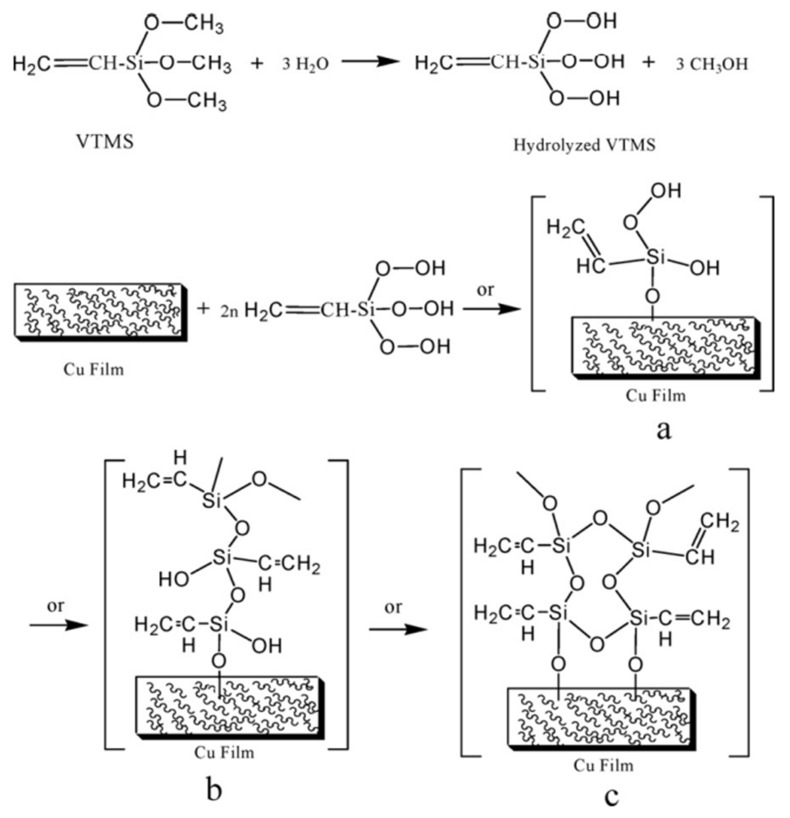
Schematic illustration of the formation of the superhydrophobic film on the copper interface. (**a**) The condensation reaction between the oxidized copper surface and silanol. (**b**) The horizontal condensation reaction among silanols. (**c**) The form of grafted polysiloxane. Reprinted with permission from Ref [66]. Copyright 2012 Elsevier.

**Figure 13:**
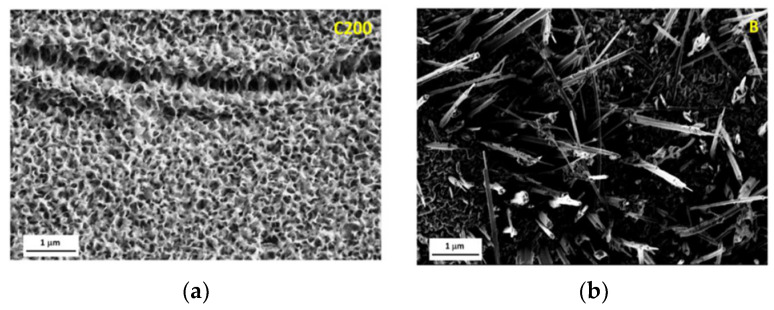
Electron microscopy images of samples treated at 200 °C (**a**) and 400 °C (**b**). Reprinted with permission from Ref [67]. Copyright 2015 Elsevier.

**Figure 14:**
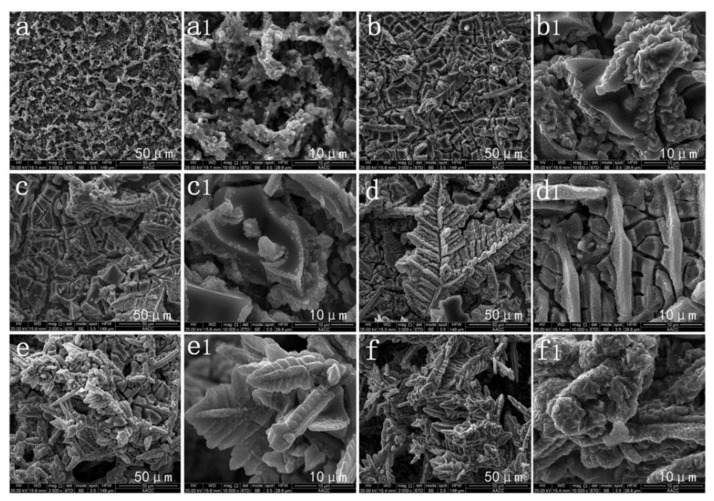
The morphological variation of the deposited Cu coating with different immersion times: (**a**) 0 min; (**b**) 3 min; (**c**) 6 min; (**d**) 9 min; (**e**) 12 min; (**f**) 15 min; (**a1**–**f1**) are images magnified 5 times by (**a**–**f**). Reprinted with permission from Ref [68]. Copyright 2016 Elsevier.

The sol-gel is a facile, fast, cost-effective, low-temperature and pressure-operated, and environmentally friendly technique. In the sol-gel method, the sols are prepared by hydrolysis and condensation of the oxides in the presence of a solvent, and then, the sols are immersed in the solvent to fabricate the gels. The sol-gel method can create great roughness structures without using any corrosive solvent, and it is the most efficient way to fabricate amorphous or crystalline oxide coatings [64,65]. In order to obtain superhydrophobic surface coatings on metal substrates, low surface energy chemical materials and nanoscale particles are necessary to add to the gels. Fan et al. [66] reported the use of a simple sol-gel method by utilizing the hydrolysis of vinyltrimethoxylsilane (VTMS) to prepare the superhydrophobic surface on the copper wafer surface. The wafers were etched by immersing in a mixture of HF and H_2_O_2_ at room temperature before the sol-gel immersion. Figure 12 illustrates a possible reaction mechanism for the sol-gel process. Figure 12a illustrates the condensation reaction between the oxidized copper surface and silanol. Figure 12b shows the formation of the film because of the horizontal condensation reaction among silanols, and Figure 12c shows the grafted polysiloxane that formed by vertical polymerization. The obtained superhydrophobic surface had a contact angle of 158.2°. Additionally, the copper superhydrophobic surface was stable in the environment and exhibited excellent corrosion resistance. Caldarelli et al. [67] fabricated a superhydrophobic Cu surface by sol-gel, sandblasted Cu foils were immersed with an alcoholic alumina sol, followed by thermal treatment and low free energy solution dipped. The superhydrophobic Cu surface with the combination of a thin film of Al_2_O_3_ flower-like structure and the low free energy organic compound (fluoroalkyl silane) displayed excellent repellence with a water contact angle approaching 180° and sliding angle less than 4°. The optimal thermal treatment temperature should not exceed 200–250 °C in order to avoid oxidation of the Cu surface, such as the surface treated at 300–400 °C (Figure 13), microwires of CuO will appear and cause the degradation of superhydrophobicity. Xia et al. [68] successfully used chemical deposition and sol-gel method to deposit CuO/Ti0_2_ coating on the surface of Q235 steel, after modification with 1H,1H,1H,2H-Perfluorodecyl-trimethoxysilaneand (FAS-17). Figure 14 shows the images of morphology when the immersion time was larger than 6 min; the surface superhydrophobicity occurred, the dendritic micro-nano roughness structure emerged when immersion time increased to 9 min, and a superhydrophobic and superoleophobic surface with a contact angle of 160° and sliding angle of 2.5° was obtained. Somoghi et al. [69] synthesized ZnO nanomaterial coatings modified with different silane coupling agents on metal substrates (Al, Cu, and Zn) by sol-gel process, the coated ZnO materials have been successfully grafted with organic functional groups (sugar groups), and the fabricated ZnO coatings have superhydrophobic properties and excellent corrosion resistance.

### 2.3. Electrochemical Deposition

**Figure 15:**
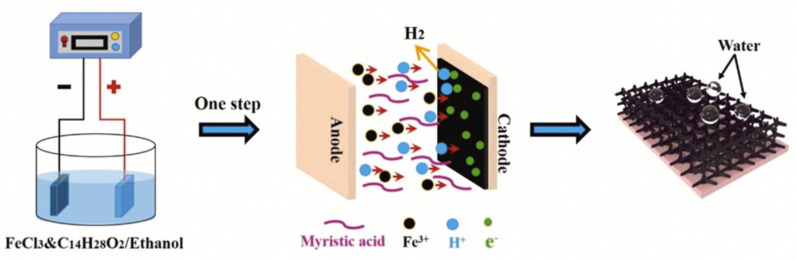
The formation process and mechanism to prepare the superhydrophobic surface on the copper surface. Reprinted with permission from Ref [72]. Copyright 2021 Elsevier.

**Figure 16:**
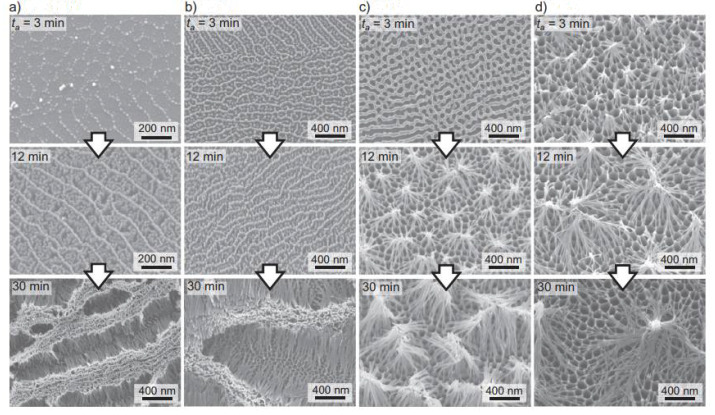
SEM images of aluminum specimens anodized in pyrophosphate solution (293K) at different voltages. (**a**) 10 V. (**b**) 25V. (**c**) 50 V. (**d**) 75V. Reprinted with permission from Ref [74]. Copyright 2018 Elsevier.

Many methods were used to fabricate superhydrophobic surfaces by generating surface roughness and modifying the low surface energy. Compared with other methods, the electrochemical deposition process can accomplish the superhydrophobic surface in just one step, which can drastically eliminate the complexity of preparing the superhydrophobic surface. Additionally, electrochemical deposition methods can be used to prepare different morphologies such as dendrites, sheets, needles, fibers, and tubes [70]. The electrochemical deposition process can be achieved by numerous methods such as anodic oxidation, polymerization, electrochemical anodization, and deposition using galvanic cells, etc. [71]. Li et al. [72] successfully fabricated a superhydrophobic film with hierarchical porous structures on the copper substrate by a simple one-step electrochemical deposition process. The formation process and mechanism to fabricate superhydrophobic surface by electrochemical deposition is shown in Figure 15. During the electrodeposition process, two copper plates worked as cathode and anode, and the electrolyte was an ethanol solution containing Fe-myristic acid. The H_2_ gas can be observed on the cathode, and with the consumption of H^+^, the CH_3_(CH_2_)_12_COO^−^ largely generated. The CH_3_(CH_2_)_12_COO^−^ reacted with Fe^3+^ to form Fe-myristic acid complex, and the complex accumulation onto Cu surface to form the superhydrophobic coating with the contact angle of 159.2° and sliding angle of 1.7° on the copper surface. Zhang et al. [73] employed a controllable one-step electrochemical deposition to manufacture an anti-corrosion superhydrophobic surface on the aluminum substrate. The electrolyte solution of the electrochemical deposition route was an ethanol solution consisting of cerium nitrate hexahydrate and hexadecanoic acid. The anode and cathode were pt plate and polished Al foil, respectively. After the electrodeposition process, the superhydrophobic surface with a static water contact angle of 167.4° and sliding angle less than 2°. Nakajima et al. [74] anodized the aluminum sheet to form nanofibrous morphology on the surface of the aluminum sheet. Figure 16 shows the surface morphology of anodized aluminum specimens at constant voltages of 10 V, 25 V, 50 V and 75 V. At lower voltages of 10 V and 25 V linear bundles consisting of numerous alumina nanofibers were formed (Figure 16a,b). At higher voltages of 50v and 75v many pyramidal bundles consisting of several alumina nanofibers were formed (Figure 16c,d). Water droplets are supported by the top of the pyramidal bundles and plenty of air is trapped in the space between the alumina bundles, leading to the formation of the superhydrophobicity. After modification with octadecylphosphonic acid, the superhydrophobic surface with a maximum contact angle of 160.1° was obtained at a voltage of 50 V for 12 min.

### 2.4. Other Methods

There are some other methods frequently used to fabricate superhydrophobic surfaces, such as chemical deposition method, vapor deposition method, template method, spray coating, and layer-by-layer assembly method. These methods are introduced briefly in this section.

Wang et al. [75] synthesized molybdenum oxide coating with a papillary bulge on the surface of Al-Mg metal by a chemical deposition method. After being modified by FAS-17, the superhydrophobic surface with a static contact angle of 160.2° and a rolling angle less than 8° was obtained. Inspired by the ability of kingfishers to fly in heavy rain without wetting their feathers, Zheng et al. [76] prepared superhydrophobic surfaces with a contact angle of 155° and a roll angle of 3° on a copper base by wire electrical discharge machining and chemical vapor deposition. Wen et al. [77] used liquid state polydimethylsiloxane (PDMS) one-step vapor deposition method to uniformly cover the circular oligomer agglomerated by irregular nanoparticles on the surface of stainless steel and prepared a superhydrophobic surface with a static contact angle of 154° and a rolling angle of 0°. Wang et al. [78] prepared a hydrophobic template by laser etching on the surface of a 6061 aluminum alloy tube. Taking the prepared 6061 aluminum alloy tube as a template and polydimethylsiloxane (PDMS) as the carrier, the superhydrophobic flexible tube with a static contact angle of 162.8° was finally obtained. Dessouky et al. [79] sprayed a polymer containing functionalized silica onto the copper surface to obtain a superhydrophobic copper surface with a contact angle of 156.9° and a rolling contact angle of less than 5°. Jiang et al. [80] assembled hydrophobic groups onto the stainless-steel mesh to form layered micro and nanostructures, a superhydrophobic stainless steel mesh with a contact angle of 159° and a rolling angle of 4° was obtained. 

## 3. Superhydrophobic Surface Drag Reduction

There are two main reasons for the drag reduction of the superhydrophobic surface: the extremely low surface energy and the effect of surface tension which reduces the contact between the liquid and the solid wall surface. We know that the maximum static friction between two solid surfaces is generally greater than the kinetic friction. The frictional force of a liquid droplet moving laterally on a solid surface was investigated by Gao et al. [81] and showed that the lateral adhesion force between liquid and solid interfaces can be divided into two states, static and dynamic, similar to solid–solid friction, and this conclusion applies to liquids with different polarities and surface tensions on smooth, rough, and structured surfaces. Liquid–solid friction is dominated by contact line friction, with interfacial friction only playing a secondary role. Conversely, contact line friction does not affect the liquid–liquid interface, as in the case of droplets on lubricated surfaces. The simulation and experimental results show that the superhydrophobic surface can retain an air film between the liquid and the solid surface. The surface minimizes the water–solid contact area, significantly reducing the frictional drag between liquid and the solid [82,83], in the superhydrophobic solid–liquid interface formed between free shear flow, solid–liquid interface shear stress is greater than the ultimate shear stress of liquid, the liquid in the superhydrophobic surface flow wall of the fluid velocity is not zero, hence boundary slippage.

The theory of liquid boundary slip was first proposed by Navier in 1823; that is, when the liquid flowing through the solid surface moves relative to the solid surface, the liquid slips relative to the solid surface. In Navier’s theory, the boundary slip velocity is proportional to the tangential shear stress of the solid–liquid interface, and the slip length is numerically equal to the distance between the theoretical no-slip boundary and the point where the fluid extends along the solid surface at a velocity of zero. Surface roughness and surface wettability are the main factors affecting the slip length [84]. In recent years, researchers have conducted a lot of simulation and experimental studies on the two main factors affecting slip and found that boundary slip is the key to the drag reduction effect of the superhydrophobic surfaces. The relationships between contact angle, wettability, and slip length are studied by the molecular dynamics, the large eddy simulation turbulence model, and the finite volume method. The results show that the higher contact angle leads to greater slip, which can effectively reduce friction and produce a drag reduction effect on the superhydrophobic surface [85,86,87,88,89]. The experimental method also confirms that the obvious velocity slip can be measured on the superhydrophobic surface and that the velocity slip is the main reason for the drag reduction of the liquid on the superhydrophobic surface [90,91,92]. The main factors affecting drag reduction of superhydrophobic surfaces are wettability, surface morphology, flow rate, and fluid viscosity. 

### 3.1. The Effects of Surface Wettability on Drag Reduction 

**Figure 17:**
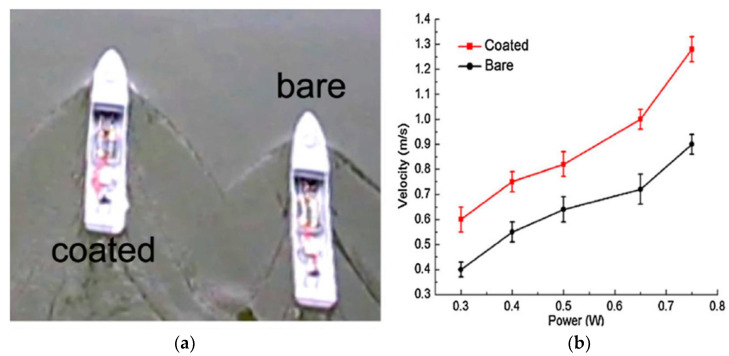
(**a**) Sailing experiment at a power supply of 0.5 W; (**b**) velocity of bare/coated model versus power supply. Reprinted with permission from Ref [94]. Copyright 2018 Elsevier.

**Figure 18:**
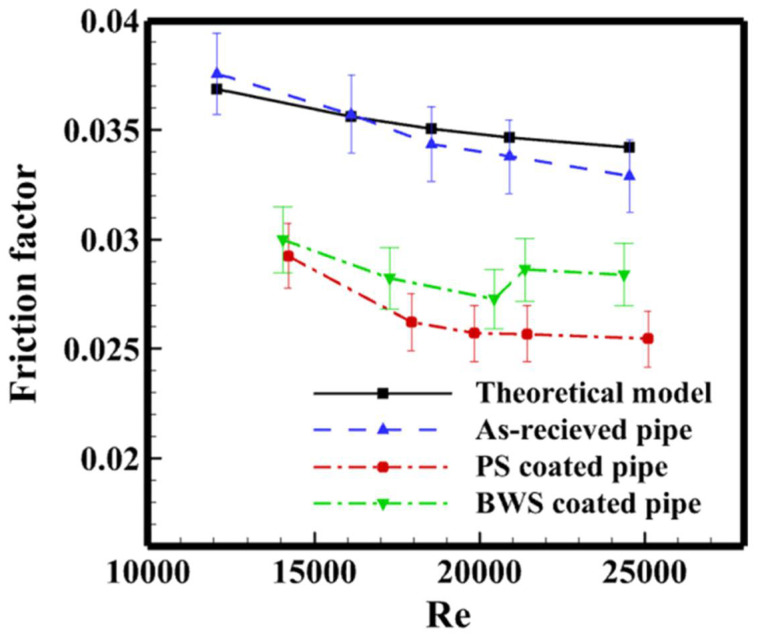
The friction factor at different Reynolds numbers. Reprinted with permission from Ref [95]. Copyright 2020 Elsevier.

The wettability of the surface has a great influence on the drag reduction effect of the surface. The experimental study showed that the drag reduction rate on the superhydrophobic surface is about 10% compared to the hydrophilic surface. Ayan et al. [93] conducted drag reduction experiments on the superhydrophilic, original, and superhydrophobic aluminum surface. The results displayed that the drag reduction increases with increasing shear Reynolds numbers, and the drag reduction on the heated superhydrophobic surface is up to 67%, with the shear Reynolds number increasing from 0.8 × 10^4^ to 3.2 × 10^4^. This is because of the stable Leidenforst vapor film and the air pockets sandwiched between roughness structures on the superhydrophobic surface. Wang et al. [94] studied the drag reduction performance through a via sailing test and rotary disc test. The controllable wettability surfaces with variable contact angles ranging from 94° to 159° were fabricated by spray coating the suspension with poly (methyl methacrylate) acrylic (PMMA) and hydrophobic nanoscale silica on steel substrates. The via sailing test results are shown in Figure 17. The average speed of the superhydrophobic coating ship model is significantly higher than that of the uncoating that the superhydrophobic surface could drag reduction effectively under low flow conditions; however, the drag force increases on the superhydrophobic surface for intensely turbulent flows. Pakzad et al. [95] fabricated superhydrophobic surfaces with PDMS and beeswax modified on the surfaces, the contact angles of 154.6° and 153.3°, respectively. The drag reduction tests on superhydrophobic surfaces were conducted, and it was when the Reynolds number was 20,000; as shown in Figure 18, the drag reduction rates on superhydrophobic surfaces with PDMS and beeswax coating were 24.62% and 19.37%, respectively. Vakarelski et al. [96] elucidated the influence of the Leidenfrost effect on the drag reduction of the superhydrophobic and hydrophilic spheres under free-falling in water. When the superhydrophobic sphere at a temperature of ~150 °C fell freely in 95 °C water, and ~300 °C in 85 °C water, there was a stable vapor layer always covered on the surface, which can promote a drag reduction of more than 75%, and the threshold thickness of the water vapor layer is related to the viscous boundary thickness. The critical transition temperature for the hydrophilic sphere to reduce drag was different from the static Leidenforst temperature. The drag reduction effect of the hydrophilic sphere is suddenly changed, and the drag coefficient of the superhydrophobic sphere is smaller than that of the hydrophilic sphere. Yu et al. [97] investigated drag reduction and slip flow of the superhydrophilic and superhydrophobic microtubules in the laminar flow. The Poiseuille number on the superhydrophobic microtubes surface was less than on that of the superhydrophilic one. As the Reynolds number is less than 900, the pressure drops and slip length on the surface of superhydrophobic microtubules decreases with the Reynolds number increasing, and a maximum drag reduction of 31% is obtained. As the Reynolds number was greater than 900, the pressure drops and slip length reached a relatively stable value of about 8%.

### 3.2. The Effects of Surface Morphology on Drag Reduction

**Figure 19:**
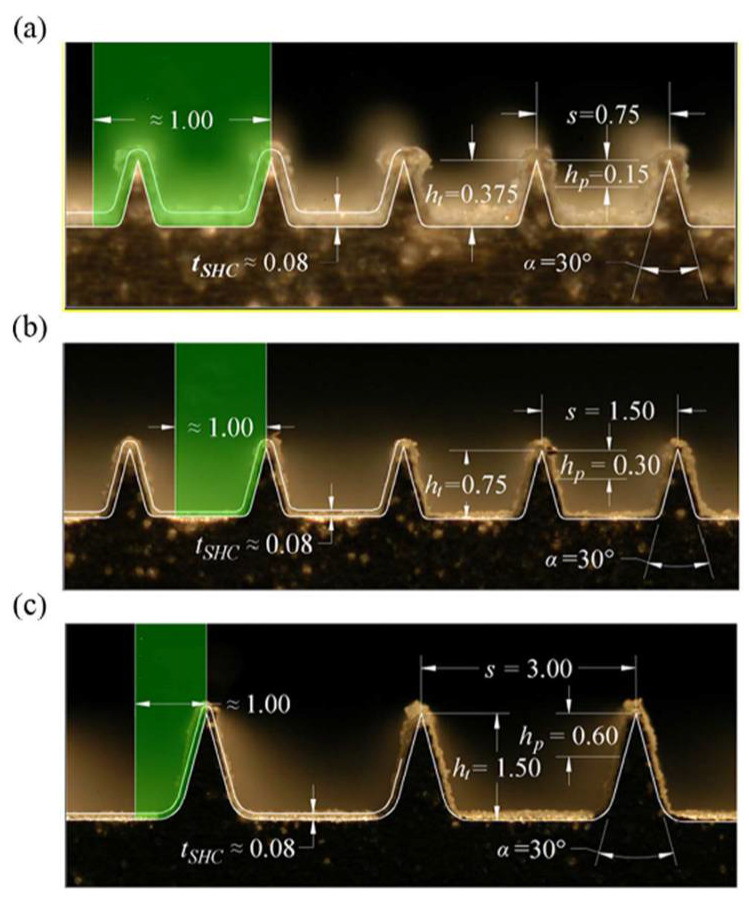
Images of the cross-section of the riblets. (**a**) s^+^ = 8.6; (**b**) s^+^ = 17.3; (**c**) s^+^ = 34.6. Reprinted with permission from Ref [100]. Copyright 2018 Elsevier.

Cui et al. [44] employed the lattice Boltzmann method to investigate the influence of surface wettability and roughness on the channel flow. A very thin vapor film occurred between the fluid and the superhydrophobic wall for the flow of the smooth surface; the pressure drop reduced as the gas/liquid interface and a slip velocity formation. For rough surfaces, the liquid sweeps through the grooves on the superhydrophobic surface, reducing the liquid–solid contact area and friction, thus achieving the drag reduction on the superhydrophobic surface. Creating rough structures on the hydrophobic surfaces has a positive effect on surface drag reduction; however, it displays a negative effect on hydrophilic surfaces. Cheng et al. [98] explained the drag reduction and heat transfer performance on the straight microchannel superhydrophobic surface with square posts, square holes, transverse and longitudinal grooves by numerical simulations method. With the increased shear-free fraction, the drag reduction and heat transfer properties of the four geometries decreased. Superhydrophobic surfaces with longitudinal have the lowest drag reduction and heat transfer, and the superhydrophobic surface with the transverse grooves has the highest. Under the condition of high shear fraction or high Reynolds number, the drag reduction and heat transfer performance on the superhydrophobic surface with square posts is better than those with longitudinal and transverse grooves. Koopaee et al. [99] examined the performance of different superhydrophobic micro post arrangements with aligned and staggered patterns under laminar flow conditions with relative module widths of 0.01, 0.1, and 1, cavity fractions of 0.1, 0.3, 0.5, 0.7, and 0.9, and Reynolds numbers of 10 and 100. The friction resistance on the staggered structure is higher than that of the aligned structure, and the staggered and aligned superhydrophobic channels have a better drag reduction effect than the traditional microchannels. The overall microchannels performance of the staggered structure is better than that of the aligned structure, and the performance is enhanced significantly when the relative modulus width is higher. Rowin et al. [100] evaluated the surface performance of different riblet superhydrophobic coatings by the planar particle image velocimetry (PIV) method. The drag reduction on the smooth and superhydrophobic surface with different riblet structures is shown in Table 1, and the images of the cross-section with different riblet structures are shown in Figure 19. The drag reduction rates on the smooth surface are 4.8% and 7.5%, with riblet tip spacing of s^+^ = 8.6 and 17.3, respectively, and the surface drag increases by 9.0% when the riblet tip spacing s^+^ = 34.6. The drag reduction rate of the surface with s^+^ = 8.6 and 17.3 increases by 1.2% and 2.6%, respectively. For the superhydrophobic surface with s^+^ = 34.6, the drag reduction rate increases by 10.2% from −9.0% (smooth surface) to 1.2%. The increase in drag reduction rate depends on the riblet tip spacing, the drag reduction on the surface is not affected by the superhydrophobic coating for smaller riblet surfaces of s^+^ < 10 and s^+^ = 17.3. For the large spacing of s^+^ = 34.6, the reason for drag reduction is that the turbulent fluid enters the riblet groove and contacts the inner wall of the groove. 

### 3.3. The Effects of Flow Rate on Drag Reduction

Wang et al. [101] experimentally studied the drag reduction performance of the superhydrophobic surface and showed that the drag reduction effect only occurred under weak flow conditions. Lv et al. [102] investigated the drag reduction and heat transfer characteristics of water flowing through the superhydrophobic tube surface. When the Reynolds numbers increase from 3000 to 11,000, the drag reduction rate of the superhydrophobic surface increases from 8.3% to 17.8%, and the drag reduction effect of superhydrophobic surfaces increases with the increase of Reynolds number and the decrease of pipe diameter. The superhydrophobic pipe shows a better drag reduction than that of the smooth pipe at the same Reynolds number. Moaven et al. [103] indicated that the drag reduction on the TiO_2_ superhydrophobic coating reached 30% and 15% in the laminar and turbulent flow, respectively. Tuo et al. [104] prepared the superhydrophobic surface on the aluminum foil; when the flow velocity was 2–5 m/s, the drag reduction rate was able to reach 20–30%. Wang et al. [101] prepared a superhydrophobic surface with a contact angle of 153.5° on the aluminum substrate. At a high shear rate, the drag reduction rate reaches 48.7%. Zhang et al. [105] proposed a novel method to fabricate 3D flow-like micro-and nanostructure films on the aluminum foil, conducted experiments on liquid/solid friction drag, and estimated the drag reduction performance of surfaces with different adhesion properties, the drag reduction rate could reach 20% to 30% at low velocity. Daniel et al. [106] measured the dissipation force of droplets moving at different velocities ranging from 0.01 to 1 mm/s on a micro- or nanostructured superhydrophobic surface, a plane grafted with a “liquid-like” polymer brush, and a lubricated surface using a cantilever force sensor with submicron accuracy. The results show that the minimum force required to move droplets on superhydrophobic surfaces and planes grafted with “liquid-like” polymer brushes is 4 and 5 μN, respectively, while for lubricated surfaces, the minimum force is 0. The dissipative force on superhydrophobic surfaces is independent of velocity, but for flat and lubricated surfaces, the dissipative force is nonlinearly related to velocity.

### 3.4. The Effects of Fluid Viscosity on Drag Reduction

Modak et al. [107] investigated the slip and drag reduction of the superhydrophobic steel balls in the creeping flow regime (Reynolds number < 0.1). The steel superhydrophobic balls ranged from 3 mm to 11.96 mm were fabricated by chemical etching. The contact angles of the superhydrophobic surface in the water, golden syrup, and honey were 157 ± 2°, 155 ± 2°, and 151 ± 2°, respectively. As shown in Table 2, the slip length of the superhydrophobic balls in the golden syrup is 178 μm~133 μm, and that is 74 μm~24 μm in the honey, and the slip length decreases with the increase of the diameter of the ball. The drag reduction rate of the superhydrophobic steel ball in the golden syrup and honey is 8.386% and 4.09%, respectively. The larger the Reynolds number, the smaller the slip length and drag reduction of the superhydrophobic steel balls, and the dominant role of the viscous effect over the convective effect is also not obvious. Xin et al. [108] fabricated a superhydrophobic surface with the grid-shape micro-nano structure on Ti-6Al-4V (TC4) alloy by laser micro-scanning method and measured the slip length of pure water (0.89 mPa) and glycerin-water solution (2.3 mPa). In the water, the average slip lengths on the hydrophilic and superhydrophobic surfaces are 0.44 μm and 17 μm, respectively. When the working liquid is 30% glycerin-water solution., the average slip length is 3 μm and 40 μm, respectively. The superhydrophobic TC4 alloy surface can effectively reduce the drag, on which the slip length with 30% glycerin-water solution is more than two times of pure water. According to the theoretical results estimated by Russian scientist Vinogradova [109] in 1995, if the superhydrophobic surface is covered with an air cushion of thickness δ between the solid and liquid, the slip length is only related to the viscosity of the working liquid, *b = δ*(*μ*_1_/*μ*_g_ *−* 1), where *μ*_1_ is the viscosity of the working fluid and *μ*_g_ is the viscosity of the air cushion. When the air-cushion thickness is constant δ, the ratio of slip length is the ratio of the viscosity of working fluids and the air. Sarkiris et al. [110] studied the motion characteristics of water and glycerol droplets on hydrophobic and superhydrophobic surfaces with different size topologies, measured the velocity and acceleration of the droplet motion, and calculated the frictional force exerted by the surface on the droplet during motion. The results show that the frictional force on the superhydrophobic surface is reduced by order of magnitude compared to that on the hydrophobic surface. The threshold force required to initiate droplet motion is higher than the threshold force at the beginning of droplet motion. With increasing roughness, the minimum frictional force to initiate the motion of a 20 μL droplet is 15–20 μN for water and 21–28 μΝ for glycerol. At the same air velocity, the acceleration of the water droplet is 400–500 mm/s greater than that of the glycerol droplet by 40–60 mm/s, owing to the combined effect of the higher hysteresis of the glycerol droplet and the larger contact line.

## 4. Boiling Heat Transfer on the Superhydrophobic Surface

Boiling heat transfer is known as one of the most effective cooling methods for high heat flux applications due to the great latent heat of the working fluidsand frequently occurs in heat transfer equipment. The characteristics of boiling heat transfer are characterized by the heat transfer coefficient and critical heat flux. The effect of surface wettability and morphology structures on the boiling heat transfer was investigated. The performances of boiling heat transfer on the smooth surface and the superhydrophobic surface are completely different, and the heat transfer coefficient on a superhydrophobic surface is higher than on a smooth surface [43].

**Figure 20:**
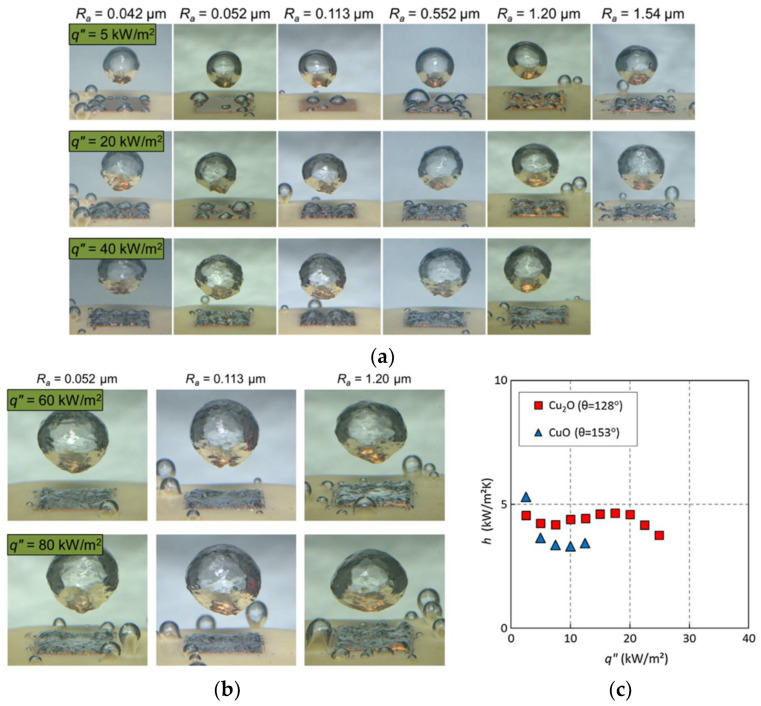
(**a**) Images of bubble nucleation and departure in nucleate and transition boiling regimes. (**b**) Images of bubble nucleation and departure in film boiling regime. (**c**) The change of heat transfer coefficient with the heat flux. Reprinted with permission from Ref [111]. Copyright 2018 Elsevier.

**Figure 21:**
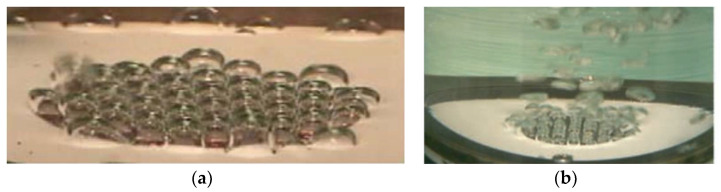
Behavior of boiling bubbles on the super-water-repellent (SWR) surface (**a**) and polytetrafluoroethylene (PTEE) surface (**b**). Reprinted with permission from Ref [112]. Copyright 2012 World Scientific Publishing Company.

**Figure 22:**
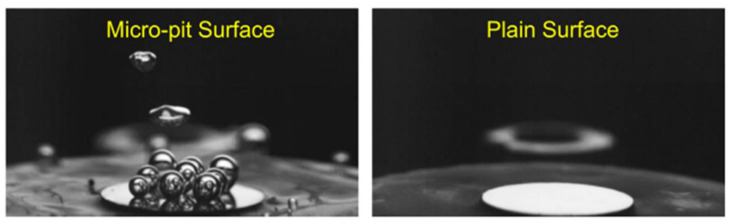
Boiling images on the micro-pit surface and plain surface with the heat flux of 3 W/cm^2^. Reprinted with permission from Ref [113]. Copyright 2020 Elsevier.

The influences of surface roughness on pool boiling heat transfer performance were investigated by Kim et al. [111]. They found the critical heat flux on a hydrophobic surface is lower than those hydrophilic surfaces. The critical heat flux on the smoothest surface (average roughness (Ra) is 0.042 µm) is about 16 times lower than on a hydrophilic one. Figure 20 shows the bubble nucleation formed and departed from the hydrophobic surface. In Figure 20a, at the heat flux of 20 KW/m^2^, the boiling surface is covered with vapor bubbles at the Ra = 1.54 µm, while the surfaces are not covered at the average roughness below 1.2 µm. At the heat flux of 40 KW/m^2^, there two surfaces (Ra = 0.552 and 1.2 µm) arrived at the film boiling region. A large number of bubbles enhances the boiling heat transfer coefficient initially on the hydrophobic surface and then rapidly weakens with the increase of the heat flux. An interesting phenomenon shown is that the boiling heat transfer coefficients maintained at 1 KW/m^2^ are unaffected by the average roughness of the heated surface. Thus, the surface wettability is not the primary factor for heat transfer of the film boiling regime. As shown in Figure 20b, all the roughness surfaces are entirely covered by vapor blanketing and have the same boiling heat transfer coefficients. It can be seen from Figure 20c, the boiling heat transfer coefficient on a superhydrophobic surface is higher than on a hydrophobic surface at the beginning heat flux of 2 KW/m^2^, while it decreases as heat flux increases. In order to investigate the performances of boiling heat transfer on the copper surface with the super-water-repellent (SWR) coating and the TiO_2_ hydrophobic surface with polytetrafluoroethylene (PTFE) coating, the experiments of pool boiling heat transfer were carried out [112]. The vapor bubbles were generated with uniform size on both treated SWR and PTFE surfaces at the low superheat of 1.5 K and 2.7 K, respectively. As shown in Figure 21, the vapor bubbles easily separate from the hydrophobic surfaces, and the superheat of bubble nucleation on the treated hydrophobic surfaces is lower than that for the original copper surface. The nucleate boiling heat transfer on the treated hydrophobic surfaces was raised seven times in contrast to that on the original copper surface. In order to enhance the nucleate boiling heat transfer coefficient and critical heat flux (CHF) on the pool boiling, the microscale pits were fabricated on the plain stainless-steel surface [113]. The boiling images on the micro-pit surface and the plain surface are exhibited in Figure 22; there are vapor bubbles formed on the micro-pit surface, while no vapor bubble is generated on the plain surface at the same heat flux of 2.5 W/cm^2^. This indicates that the wall superheats on the micro-pit surface at the onset of nucleate boiling are lower than that on the plain surface, and the nucleated boiling heat transfer coefficient and critical heat flux all promoted significantly on the micro-pit surface. The pool boiling heat transfer with different structures of the micro-pit was investigated, and the optimal heat transfer coefficient and critical heat flux were 70.0 KW/m^2^K and 165.7 W/cm^2^, which improved 58.5% and 33.7% compared with the plain surface, respectively. The influences of surface microstructure with ten rib and post geometries on the pool boiling heat transfer performance were studied [114]. The cavity fraction (from 0.5 to 0.98), pitch (from 8 µm to 40 µm), and feature height (from 4 µm to 15 µm) were used to define the surface microstructures in geometry. On rib patterned superhydrophobic surfaces, the transition from nucleate boiling to film boiling is primarily dependent on the cavity fraction, and with the cavity, fraction increases the temperature that transition from nucleate to film boiling is lower than on post patterned surfaces. The transition temperature increased with the microstructure feature height increasing from 4 µm to 15 µm. On post patterned superhydrophobic surfaces, the cavity fraction and pitch are not the influence factors of the pool boiling heat transfer, and these post microstructures are more restrained nucleate boiling. However, the heat flux is unaffected by the surface microstructures once stable film boiling has arrived.

**Figure 23:**
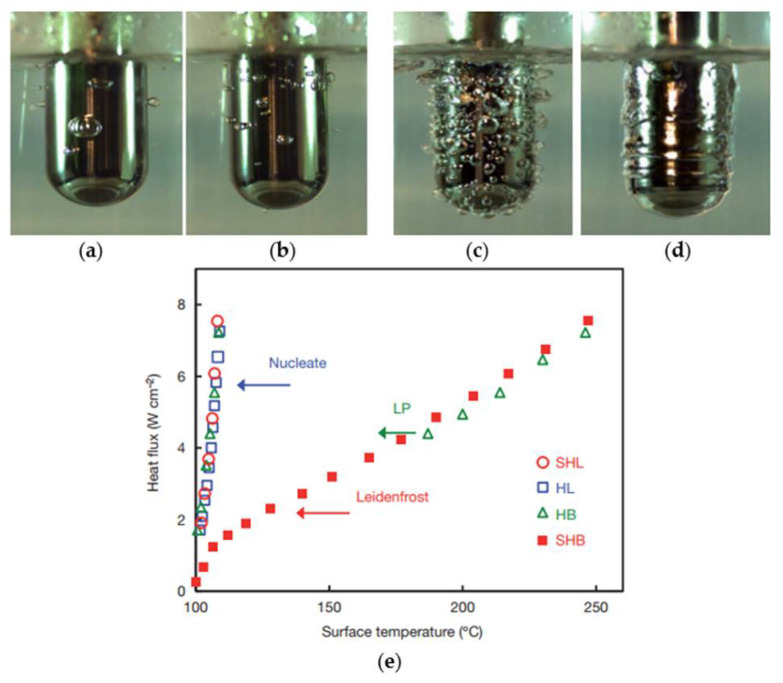
The heat transfer images on the superhydrophilic surface (SHL) (**a**), hydrophilic surface (HL) (**b**), hydrophobic surface (HB) (**c**), and superhydrophobic surface (SHB) (**d**). (**e**) The relation curve of heat flux and surface temperature. Reprinted with permission from Ref [115]. Copyright 2012 Springer Nature.

**Figure 24:**
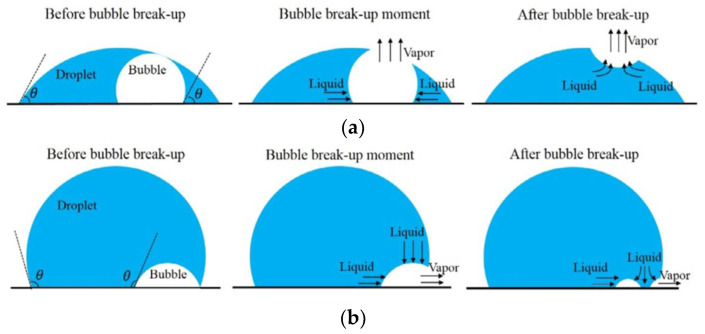
The process of bubble breaking within a droplet on the hydrophilic surface (**a**) and the hydrophobic surface (**b**). Reprinted with permission from Ref [117]. Copyright 2020 Elsevier.

The performances of boiling heat transfer on the superhydrophilic, hydrophilic, hydrophobic, and superhydrophobic surfaces were experimentally studied [115]. The surfaces are all dipped in water that is closed to saturation, and all heat transfer is in the nucleate boiling state with the same heat flux of 8.0 W/cm^2^ (less than the critical heat flux). Based on the experimental results, the surface temperature of bubbles generated on the superhydrophobic surface is 210 °C higher than 106 °C for the superhydrophilic and hydrophilic surfaces. As can be seen from Figure 23a–c, the vapor bubbles were more easily generated on the hydrophobic surface than on the superhydrophilic and hydrophilic surfaces at small superheat, while for the superhydrophobic surface, a maintained vapor layer was generated around the surface (Figure 23d). The characteristics of boiling heat transfer on the four treated surfaces are shown in Figure 23e; nucleate boiling is maintained on superhydrophilic and hydrophilic surfaces with the lower surface temperature at a steady state. However, the superhydrophobic surface maintains a vapor layer in the Leidenfrost regime in all cases of surface superheating. For hydrophobic surfaces, it is only possible to maintain the Leidenfrost vapor regime by superheating the cylinder to 350 °C prior to dipping and using maximum power heating during dipping. When the surface temperature was reduced below 170 °C, the Leidenfrost vapor layer collapsed and transitioned to nucleation boiling. The performances of boiling heat transfer on superhydrophilic, hydrophilic, and hydrophobic surfaces are better than that of superhydrophobic surfaces. Betz et al. [116] investigated the boiling heat transfer performances on superhydrophilic and superhydrophobic surfaces as well as on superbiphilic surfaces with superhydrophilic and superhydrophobic regions juxtaposed. The results indicated that the nucleate boiling easily occurred on the superhydrophobic surface at small superheat, and the largest heat transfer coefficients and critical heat flux occurred on the biphilic surfaces at small superheat as the hydrophilic regions inhibit the generation of vapor layers. Moreover, the superbiphilic surfaces have heat transfer coefficients up to three times higher than state-of-the-art nano surfaces. The above-mentioned heterogeneous surfaces were also used for the study of droplet nucleate boiling [117]. As shown in Figure 24, the bubble inside the droplet breaking and is completely expelled at the top of the droplet on the hydrophilic surface, and breaking at the edge of the droplet on the superhydrophobic surface with some vapor will be residue in the droplet. At a superheat of 20 K, lower nucleate boiling temperature and 48.08% higher heat transfer rate on the hydrophobic surface compared to the hydrophilic surface, and the heat transfer rate on the heterogeneous hydrophobic surface is 381.25% higher than a hydrophilic surface. The increase of superhydrophobic regions on heterogeneous hydrophilic surfaces further enhances the heat transfer of droplet nucleate boiling. The heat transfer performances of flow boiling in micro pin-fin arrays were also affected by surface wettability. Qin et al. [118] conducted experiments to research the flow boiling heat transfer on the four treated micro pin-pit surfaces (circular, ellipse, diamond, and triangle). The flow boiling heat transfer performance of superhydrophobic or hydrophobic surfaces with diamond microarrays is better than the bare copper surface in film boiling, transition, and nucleate boiling regions. The pressure drop on the hydrophobic surface is 90% smaller than the bare copper surface for the mass flux of 215 kg/(m^2^·s). The performance of flow and thermal on the surfaces with diamond microarrays is better than the shapes of circular, ellipse, and triangle. Moreover, the heat transfer coefficient of diamond microarrays is 76% greater than that of triangles. The flow boiling heat transfer characteristics of copper substrates with carbon nanotube (CNT) and diamond coating were studied by Kumar et al. [119]. The results demonstrated that the nucleation density on the CNT and diamond coated hydrophobic surfaces was higher than that of sandblasted copper surface, and the critical heat flux on the CNT-coated hydrophobic surface were improved by 21.6%, 14.28%, and 6.69%, respectively, to the blasted copper surface for the mass flux of 283, 348, and 427 kg/(m^2^·s), respectively. 

The boiling heat transfer performance on a superhydrophobic surface is better than on the hydrophobic and hydrophilic surfaces at low superheat, and with the increased superheat, the heat transfer performance degradation. Vapor bubbles are easily formed and depart on superhydrophobic surfaces, the superheat of bubble nucleation is lower than that of hydrophobic and hydrophilic surfaces, and the heat transfer performance of nucleated boiling is significantly better than that of hydrophobic and hydrophilic surfaces. The transition from nucleate boiling to film boiling on the superhydrophobic surface is easier than that of the hydrophilic surface with the increasing superheat. The microstructures on the superhydrophobic surface have a dramatic effect on the nucleate boiling heat transfer; however, the boiling heat transfer is not affected by the microstructures when it enters a stable film boiling.

## 5. Condensation Heat Transfer on Superhydrophobic Surfaces

**Figure 25:**
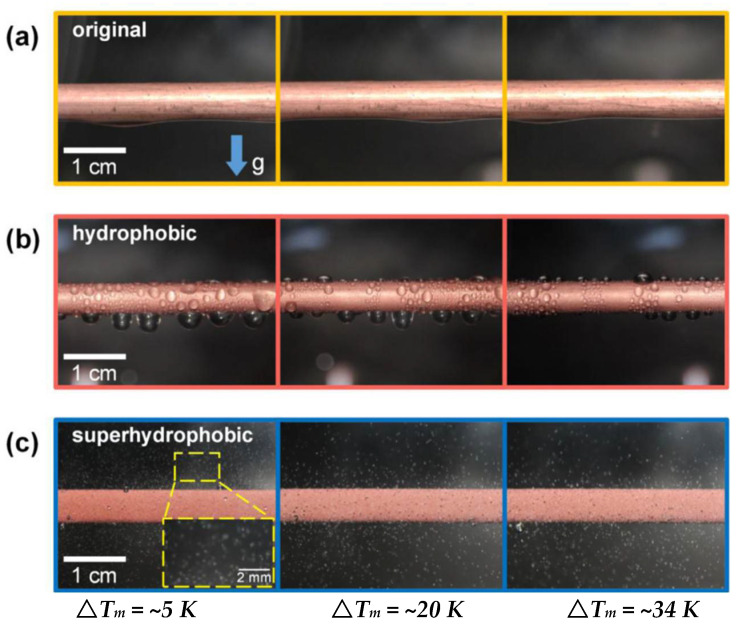
The condensation behaviors on the original (**a**), hydrophobic (**b**), and superhydrophobic surfaces (**c**) with the subcooling increasing. Reprinted with permission from Ref [122]. Copyright 2020 Elsevier.

Condensation is a ubiquitous phenomenon that is often applied in various industrial processes. It is well known that the heat transfer efficiency for dropwise condensation is almost an order of magnitude higher than filmwise condensation [120]. In general, hydrophobic surfaces can facilitate dropwise condensation, while hydrophilic surfaces can induce filmwise condensation. The condensation heat transfer performance of pure vapor during drop condensation on a superhydrophobic surface was reported by Bisetto et al. [121]. The results show that increasing the vapor velocity helps maintain high droplet mobility. In the dropwise condensation mode, the superhydrophobic surface showed better condensation performance than the untextured oxidized surface and worse performance when transitioning to filmwise condensation. The condensation behaviors on original, hydrophobic, and superhydrophobic surfaces are shown in Figure 25 [122]. The stable filmwise condensation occurred on the original hydrophilic surface, which can be seen in Figure 25a; there is no transition in condensation mode with increasing the subcooling to ~34 K. As displayed in Figure 25b, a stable dropwise condensation model is maintained throughout the subcooling increase. In Figure 25c, the coalesced small droplets can be observed to jump off the superhydrophobic surface spontaneously. Thus, the heat transfer on the superhydrophobic surface is significantly better than those on the original and hydrophobic surfaces at low subcooling. However, the heat exchange deterioration at high subcooling as the droplet jumping disappeared with the subcooling greater than the critical value. 

**Figure 26:**
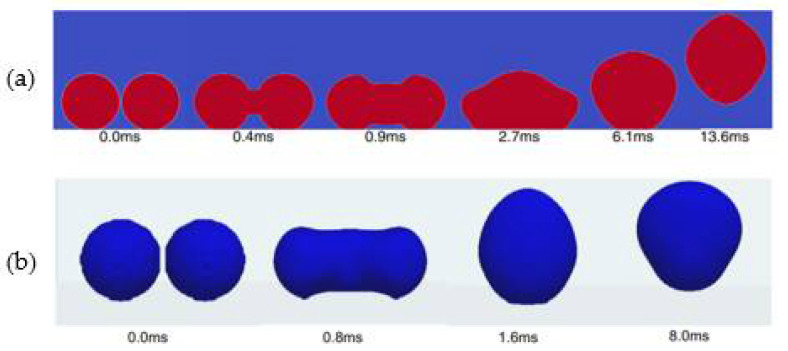
The droplets coalescence and jumping phenomena on the superhydrophobic surface. (**a**) 2D lattice Boltzmann method. (**b**) 3D volume of fluid method. Reprinted with permission from Ref [123]. Copyright 2016 Elsevier.

**Figure 27:**
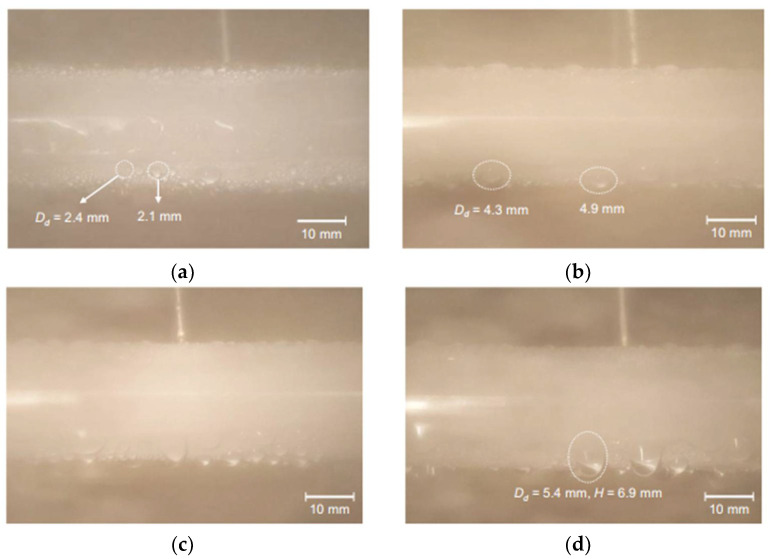
Condensation phenomena. (**a**) Dropwise condensation phenomenon. (**b**) Flooded condensation phenomena (departing frequency: 19.6 drops/min). (**c**) Flooded condensation phenomena (departing frequency: 19.6 drops/min). (**d**) Attached condensation phenomena (departing frequency: 8.1 drops/min). Reprinted with permission from Ref [124]. Copyright 2019 Elsevier.

**Figure 28:**
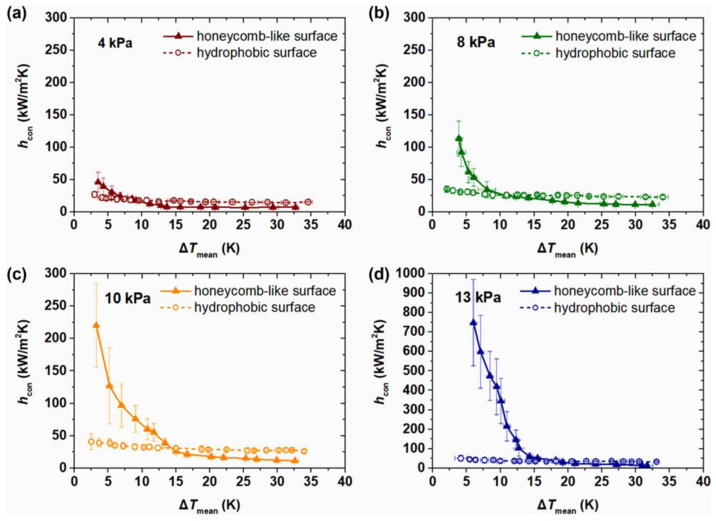
The condensation heat transfer coefficient on the honeycomb-like superhydrophobic and hydrophobic surface. (**a**) 4 kPa. (**b**) 8 kPa. (**c**) 10 kPa. (**d**) 13 kPa. Reprinted with permission from Ref [125]. Copyright 2021 Elsevier.

**Figure 29:**
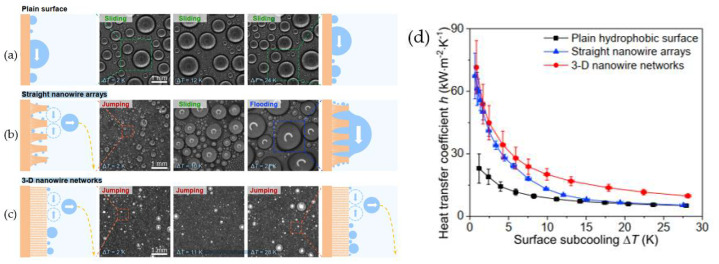
The dynamic behavior of droplets on (**a**) plain hydrophobic. (**b**) straight superhydrophobic nanowire arrays. (**c**) 3D superhydrophobic nanowire networks surfaces. (**d**) The condensation heat transfer coefficient is a function of surface subcooling. Reprinted with permission from Ref [127]. Copyright 2018 Elsevier.

Khatir et al. [123] used 2D lattice Boltzmann and 3D Volume of Fluid methods (shown in Figure 26a,b) to analyze the coalescence of droplets and jumping phenomena on the superhydrophobic surface. The merged droplets jump upward off the surface, and the diameter of the droplets is larger than the initial droplets. Attributed to the smaller contact area between the merged droplet and the hydrophobic surface, the energy required to dewet the surface is less, and jumping velocity on the superhydrophobic surface is higher than those hydrophobic and hydrophilic surfaces. Therefore, the merged droplet is more easily detached from the superhydrophobic surface, leading to enhanced condensation heat transfer performance. Ji et al. [124] investigated the condensation heat transfer performance on superhydrophobic and bare aluminum surfaces. The visual experiment of dropwise, flooded, and attached condensation was conducted, as shown in Figure 27. The dropwise condensation could be observed in Figure 27a; the droplets with diameters less than 3 mm were evenly distributed on the superhydrophobic surface. In Figure 24b,c flooded condensation occurred on the superhydrophobic surface with the droplet departing frequency of 19.6 and 38 drops/min, respectively. The diameter of hemispherically shaped droplets is more than 4 mm greater than the dropwise condensation. It can be seen in Figure 27d that under attached condensation, the higher and wider droplets generated compared to the flooded condensation, and the droplet departing frequency was reduced to 8.1 drops/min. As a result, the heat transfer performances of dropwise condensation and flooded condensation that on the superhydrophobic surface were better than that of the bare tube, and the condensation heat transfer coefficient improved by 105% and 16%, respectively. However, the heat transfer performance of attached condensation degradation of about 20% compared to the bare tube. Therefore, the attached condensation phenomenon should be avoided in the application. The performances of steam condensation heat transfer on the superhydrophobic surface with scalable honeycomb-like microporous under different pressures were investigated by Zhang et al. [125]. The condensation heat transfer coefficient on the honeycomb-like superhydrophobic and hydrophobic surfaces was displayed in Figure 28. The condensation heat transfer on the honeycomb-like superhydrophobic surface is better than hydrophobic and hydrophilic surfaces at low subcooling under all pressures. As shown in Figure 28a,c, the highest condensation heat transfer coefficients on the honeycomb-like superhydrophobic surface were improved by ~50%, 300%, and over 400% compared to that of the hydrophobic surface under 4 kPa, 8 kPa, and 10 kPa, respectively. Under 13 kPa (Figure 28c), the maximum heat transfer coefficient reaches 700 kW/m^2^K. The performance of condensation heat transfer on the superhydrophobic surface degenerated at high subcooling, and even lower than that of the hydrophobic surface, the critical point of transition from dropwise to flooding model was increased from ~7 K under 4 kPa to ~17 K under 13 kPa. The condensation heat transfer on the superhydrophobic surface with Si nanowires coating was investigated by Lu et al. [126]. The result demonstrated that the droplets jumping-off frequency on the superhydrophobic surface was higher, that leading to the heat transfer coefficient on the superhydrophobic surface (88 ± 16 kW/m^2^K) being 155% and 87% greater than that of on the hydrophilic and hydrophobic surfaces, respectively. Further, the droplets can still be quickly escaped the superhydrophobic Si nanowires surface quickly at high subcooling, and the heat transfer coefficient was 18.6 ± 16 kW/m^2^K larger than those on the plain hydrophilic and hydrophobic surface. The dropwise condensation characteristics on superhydrophobic surfaces were studied by Wen et al. [127]. The morphology, coalescence, and dynamic for condensed droplets are illustrated in Figure 29. Figure 29a displayed the conventional dropwise condensation, the jumping-off droplets with a diameter ranging from a few micrometers to millimeters, and the droplet jumping-off frequency increases as the increased subcooling resulting in accelerated droplet growth rate. The droplet generation, growth, and jumping off on the straight superhydrophobic nanowire arrays surface can be seen from Figure 29b, the droplet departure diameter (<500 µm) is greater than that of on a plain surface at low subcooling of ~2 K. When the supercooling increases to 10 K, the concentrated droplet has a larger diameter (700–800 µm) and departed by gravity rather than jumping off the surface. As the subcooling increases to 21 K, large droplets formation and pin on the surface, and the condensation model is the transition to flooding condensation. The reason for this condensation phenomenon is that the nucleation occurred on top of the nanowire array and the micro-defects; with subcooling increases, a large number of nano-droplets formed and grew rapidly to fill the micro-defects, which leads to the transition of flooding condensation. Contrary to the straight nanowire arrays, Figure 29c shows the stable dropwise condensation on the 3D superhydrophobic nanowire networks surface throughout entire surface subcooling ranging from 2 K to 28 K. This is attributed to the tightly aligned 3D nanowires without micro-defects can promote suspension of droplets on the nanowires. The heat transfer coefficient on these three surfaces is shown in Figure 29d, and the biggest heat transfer coefficient is accessed on the 3D nanowire networks surface, which is 100% higher than that of on a plain hydrophobic surface. The characteristics of moist air condensation on hydrophilic and superhydrophobic surfaces were experimentally researched by Wu et al. [128]. Condensate droplet departure on hydrophilic surfaces is mainly driven by gravity, whereas on superhydrophobic surfaces, it is principally due to spontaneous droplet jumping off. The condensate droplets formed on the superhydrophobic surface are about 40–65% lower than that of the hydrophilic surface, and the heat transfer coefficient is 36% and 16% larger than that of the hydrophilic surface at relative humidity is 85% and 40%, respectively. The effects of initial wetting state and surface tilt on droplet kinetics and condensation heat transfer were investigated by Wang et al. [129] through experiments and lattice Boltzmann simulations. The heat transfer coefficients are improved by 21.1%, 49.2%, and 72.4% at the tilt angle are 30°, 60°, and 90°, respectively. When the surface subcooling is raised from 0.5 to 3.5 K, the droplet jumping frequency (tilt angle is 90°) decreases from 173 to 36 cm^−2^ s^−1^, the average diameter of the droplet increases ~300%. When the tilt angle is 30°, the critical sliding diameter of droplets is 1.5, 1.4, and 1.5 times greater than that of 90° as the surface subcooling are 5.0 K, 6.5 K, and 8.0 K, respectively. The performance of dropwise condensation heat transfer on the superhydrophobic copper surface with binary microgroove and nanocone structures was reported by Chen et al. [130]. The superhydrophobic with microgroove/nanocone structure has the best condensation performance, and the heat transfer coefficient is 82.9% higher than that of the plat hydrophobic copper surface.

**Figure 30:**
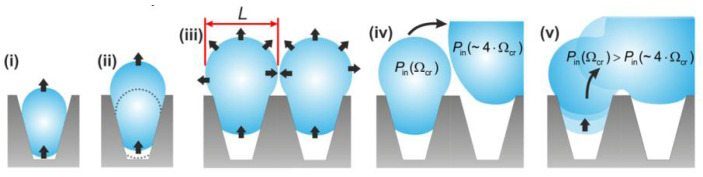
Schematic diagrams depicting the different states experienced by the droplets. Ω is the total volume of the droplet. (**i**) The droplet grows inside the microcone cavity until ΔP_in_ becomes slightly positive and starts to move outside the cavity. (**ii**) The upper meniscus contact line of the droplet reaches the top of the microcavity and the lower meniscus contact line moves upward. (**iii**) The droplet grows further to the critical upper meniscus radius of curvature R_t,cr_ = L/2 above which coalescence with the neighboring droplets occurs. (**iv**) Coalescence of a droplet Ωcr with a larger droplet of at least ~4 Ω_cr_. The average pressure P_in_(Ω_cr_) within a small droplet is much larger than P_in_(~4 Ω_cr_) within a larger droplet. (**v**) This coalescence induces a pressure difference that drags the small droplets out of the microcone cavity. Reprinted with permission from Ref [133]. Copyright 2018 American Chemical Society.

The effects of superhydrophobic surface structure, nucleation density, droplet morphology, and droplet dynamics on condensation heat transfer were reviewed by Miljkovic et al. [131]. Stretchable superhydrophobic surfaces fabricated from metal oxides are considered to be one of the most promising approaches due to their ability to form partially wetted droplets, relatively large thermal conductivity, small structural length scales, and low droplet adhesion for stable droplet hopping. Efficient droplet hopping increases condensation heat transfer, with a 30% heat transfer enhancement observed in pure vapor environments; however, these surfaces remain limited due to the occurrence of water immersion in applications. Miljkovic et al. [132] prepared scalable silanized copper oxide superhydrophobic surfaces and experimentally investigated the condensation performance of the superhydrophobic surfaces. The results indicate that the prepared superhydrophobic surfaces can achieve efficient heat transfer for hopping droplet condensation. At supersaturation levels below 1.12, the overall heat flux and condensation heat transfer coefficient are improved by 25% and 30%, respectively, compared to the state-of-the-art hydrophobic condensation surface. At supersaturation levels greater than 1.12, the flooding of the nanostructured surface leads to the formation of highly pinned Wenzel droplets, which deteriorate the condensation heat transfer coefficient by 40% compared to smooth dropwise condensate tubes. Nanostructured superhydrophobic surfaces can be used not only to enhance condensation heat transfer but also to boost atmospheric water collection and dehumidification efficiency. Sustainable condensation of small-diameter droplets on metal surfaces can enhance the thermal efficiency of condenser devices. Sharma et al. [133] employed three-dimensional laser micromachining and surface self-assembly to create a superhydrophobic surface with a regular array of microcones covered by nanostructures on a metal surface. The passive departure of droplets on this surface was achieved by the gradual coalescence of droplets generated in the microcavities formed by the microcone arrays, as shown in Figure 30. (i) The droplet grows inside the microcone cavity and starts to move to the outside. (ii) The top semilunar contact disk of the droplet reaches the top of the microcavity while the lower semilunar contact line is moving upward. (iii) The droplet grows further until the radius of curvature of the upper half-moon surface is L/2, and coalescence with neighboring droplets occurs. (iv) When a droplet (Ω_cr_) coalesces with a larger droplet (∼4 Ω_cr_). The average pressure inside the smaller droplet is greater than that inside the larger droplet. (v) This coalescence induces a pressure difference that drags the small droplet out of the microcone cavity. The droplets are then controlled to leave under vapor flow conditions, which significantly enhances the heat transfer. The synergistic effect of vapor shear and continuous dropwise condensation on the superhydrophobic copper surface resulted in a nearly 700% increase in heat transfer coefficient compared to filmwise condensation from identical, standard unstructured surfaces.

The heat transfer performance of superhydrophobic is significantly better than that of hydrophobic and hydrophilic surfaces at low subcooling. The condensation performance on superhydrophobic surfaces is different from that on normal untreated surfaces. On superhydrophobic surfaces, condensate droplets roll off the surface easily; however, on normal untreated surfaces, droplets are pinned to the surface, that causing deterioration of condensation heat transfer and then leading to the transition of filmwise condensation. Therefore, on superhydrophobic surfaces, the generation of filmwise condensation can be effectively avoided.

## 6. Conclusions

In this study, a comprehensive review of the research progress of superhydrophobic surfaces and their applications in industry and life in the last decade is presented. Super-hydrophobicity, surface wetting models, and methods for the preparation of superhydrophobicity on metal and alloy substrates are described. The excellent performance of superhydrophobic surfaces for drag reduction, boiling heat transfer, and condensation heat transfer is analyzed in detail in different parts of the paper. Superhydrophobic surfaces have been extensively applied in industry, for example, to delay the formation of ice and frost on airplanes, wind turbines, and heat exchanger surfaces, enhance the corrosion resistance of metal and alloy surfaces, improve the heat transfer properties of boiling and condensation, and so on. These applications have positive implications for energy savings and improved surface performance.

Although the application of superhydrophobic surfaces has been deeply researched, more advanced investigations are necessary in fundamental theory; for example, the fabrication of superhydrophobic surfaces still suffers from high cost, poor durability, and technical complexity. When superhydrophobic surfaces are exposed to acidic and alkaline environments, high temperatures, mechanical wear, and cyclic impacts, they tend to lose the superhydrophobic properties, so research on both durability and robustness is indispensable. The boiling and condensation heat transfer characteristics and mechanisms of superhydrophobic surfaces are different from those of normal and hydrophilic surfaces, and further theoretical and experimental studies in this aspect are necessary.

In the majority of the papers investigated, the working medium for boiling and condensing heat transfer is pure water or aqueous solutions with very low viscosity. However, in industrial applications, it is often encountered that the working fluid has a high viscosity and tends to adhere to the surface, resulting in degradation of the heat transfer coefficient. Moreover, the research on heat transfer of viscous fluids on superhydrophobic surfaces is rather little, so the possibility of superhydrophobic surfaces to reduce the flow resistance of high-viscosity fluids and improve the heat transfer efficiency is a worthwhile work in the future.

## Figures and Tables

**Table 1:** Comparison of drag reduction between superhydrophobic surface and smooth surface. Reprinted with permission from Ref [100]. Copyright 2018 Elsevier.

Surface	Drag Reduction %
s^+^ = 8.6 (smooth)	4.8
s^+^ = 17.3 (smooth)	7.5
s^+^ = 34.6 (smooth)	−9.0
s^+^ = 8.6 (superhydrophobic)	6.0
s^+^ = 17.3 (superhydrophobic)	10.1
s^+^ = 34.6 (superhydrophobic)	1.2
Flat surface (superhydrophobic)	6.9

**Table 2:** Flow drag reduction in honey and golden syrup. Reprinted with permission from Ref [107]. Copyright 2017 Elsevier.

Medium	D_p_/(mm)	ρ_s_/ (kg/m³)	υ_SM_^①^/ (mm/s)	υ_SH_^②^/ (mm/s)	Re/(10^3^)	b_s_/(μm)	Drag Reduction /(%)
Golden syrup	3.192	7801	1.427	1.557	0.263	178	8.386
	4.762	7700	3.172	3.375	0.871	174	6.005
	6.365	7800	5.671	5.590	2.083	173	4.684
	8.764	7790	10.723	11.104	5.423	167	3.437
	9.455	7790	12.479	12.867	6.809	156	3.015
	11.96	7790	19.917	20.343	13.747	133	2.094
Honey	3.192	7801	5.061	5.277	3.534	74	4.09
	4.762	7700	11.418	11.699	12.089	61	2.4
	6.365	7800	19.823	20.159	27.230	56	1.667
	8.764	7790	39.646	40.026	79.175	43	0.947
	9.455	7790	41.979	42.327	82.124	40	0.823
	11.96	7790	57.091	57.320	120.515	24	0.4

^①^ settling velocities of a smooth ball. ^②^ settling velocities of the superhydrophobic ball.

## Data Availability

Not applicable.
